# The Role of Routine Surveillance Cultures in Optimising Sepsis Management in High-Risk Patient Groups

**DOI:** 10.3390/pathogens15010082

**Published:** 2026-01-12

**Authors:** Jan Závora, Václava Adámková, Alžběta Studená, Gabriela Kroneislová

**Affiliations:** 1 Clinical Microbiology and ATB Centre, General University Hospital in Prague, 128 08 Prague, Czech Republic; 2 Department of Medical Microbiology, Palacky University, 779 00 Olomouc, Czech Republic

**Keywords:** surveillance, microbiology screening, blood culture, multiresistant bacteria, sepsis

## Abstract

Background: Sepsis remains a leading cause of morbidity and mortality, particularly when caused by multidrug-resistant organisms (MDROs). Early identification of colonising or infecting pathogens may inform initial antimicrobial selection. Surveillance cultures, providing microbiological data prior to infection onset, could guide timely and targeted therapy. This retrospective study analysed routine surveillance culture results from patients with bloodstream infection (BSI) episodes, assessing pathogen prevalence, resistance phenotypes, and concordance with specimen type in haemato-oncology (HO) and acute care (AC) settings. Methods: Data were retrieved from the institutional Laboratory Information System of the Department of Clinical Microbiology and ATB Centre, General University Hospital in Prague, covering 1 January to 31 December 2024. All positive blood cultures containing ESCAPE pathogens (excluding *Clostridioides difficile*) were reviewed. Corresponding surveillance culture records were analysed to evaluate concordance with subsequent BSI episodes. Results: In 2024, 6046 AC and 7267 HO surveillance cultures were performed; MDRO prevalence was 5% and 6.56%, respectively. ESBL-producing Enterobacterales predominated (AC 86.9%, HO 81.6%). In HO, BSI-causing Gram-negative and Gram-positive pathogens were frequently detected in rectal swabs, whereas in AC, concordance was higher with upper and lower respiratory tract samples. Rectal screening detected 100% of *E. coli* and *K. pneumoniae* BSI episodes in HO. Other specimen types showed limited concordance. Conclusions: Surveillance culture utility varies by specimen type and clinical setting. In both HO and AC units, these cultures provided valuable insights into colonisation and resistance patterns, supporting early risk stratification and guiding initial therapy in high-risk patients.

## 1. Introduction

Sepsis remains one of the most severe and life-threatening infections in clinical practice, with mortality rates rising sharply when caused by multidrug-resistant pathogens [1]. Of particular concern are bacterial species within the ESCAPE group (*Enterococcus faecium*, *Staphylococcus aureus*, *Clostridioides difficile*, *Acinetobacter baumannii*, *Pseudomonas aeruginosa*, and Enterobacterales), whose resistance mechanisms significantly limit therapeutic options. Early identification of colonising or infecting pathogens, including multidrug-resistant organisms, may support more informed selection of initial antimicrobial regimens and improve therapy appropriateness, which is associated with reduced mortality when antimicrobials are administered promptly. However, in many high-risk patients, initial antimicrobial therapy is introduced before microbiological confirmation is available, highlighting the need for reliable patient-specific predictors to guide early treatment decisions. By providing microbiological data prior to the onset of infection, surveillance cultures can play a pivotal role in guiding timely and targeted treatment strategies, potentially improving patient outcomes and mitigating the burden of antimicrobial resistance [2,3,4].

Furthermore, integration of routine screening results into clinical decision-making aligns with the principles of antimicrobial stewardship and individualised patient care [5]. Despite the critical role of microbiological diagnostics in guiding antimicrobial therapy for sepsis, substantial gaps remain in how routine screening strategies are implemented and utilised in clinical practice. Studies indicate that even when microbiology tests are ordered, a relatively small proportion of results inform antibiotic decision-making, suggesting underutilization of available diagnostic data. This underutilization represents a missed opportunity to optimise initial and early targeted antimicrobial therapy, particularly in patient populations at high risk of sepsis-related morbidity and mortality [6].

Within microbiological surveillance programmes, detection of both resistant and antimicrobial-susceptible organisms can provide clinically actionable information to guide both initial and definitive therapy, particularly in high-risk populations such as haemato-oncology patients and those admitted to intensive care units. While most surveillance protocols focus primarily on multidrug-resistant organisms due to their obvious therapeutic implications, colonisation with susceptible flora also offers valuable insight and may inform the selection and de-escalation of initial antimicrobial regimens [7]. Historically, surveillance cultures in neutropenic haematologic patients were recommended to identify a range of organisms, including common Gram-negative and Gram-positive bacteria, that preceded BSI, highlighting that knowledge of colonising susceptible microbes may anticipate subsequent invasive disease. Comprehensive surveillance capturing the full spectrum of colonising organisms, rather than resistant strains alone, may therefore improve the appropriateness of antimicrobial therapy in sepsis management within haemato-oncological and intensive care settings. Nevertheless, data systematically evaluating the relationship between routine surveillance findings and subsequent bloodstream infections in these high-risk settings remain limited [8].

In this retrospective study, the results of routine surveillance cultures from patients BSI episodes were analysed. The prevalence of pathogens—including resistance phenotypes—and their relationship to specimen type and subsequent BSI were evaluated in patients from haemato-oncology and acute care units to assess the utility of surveillance cultures in guiding timely and targeted antimicrobial therapy. The data were obtained from clinical samples of patients hospitalised at the General University Hospital in Prague, which provides centralised oncological and acute care across multiple departments.

For the purposes of this study, surveillance cultures were defined as microbiological cultures performed to detect patient colonisation rather than for diagnostic purposes. A BSI episode was defined as the isolation of a pathogen from one or more blood cultures in a patient with clinical signs of infection, lasting until microbiological and clinical resolution or a predefined time interval [9]. Sepsis was defined as a life-threatening organ dysfunction caused by a dysregulated host response to infection [10]. Multidrug-resistant (MDR) organisms were defined as non-susceptible to at least one agent in three or more antimicrobial categories [11]. Extended-spectrum beta-lactamase (ESBL) production was defined in this setting by resistance to third-generation cephalosporins and growth on selective media. Carbapenem resistance was defined as in vitro non-susceptibility to meropenem and/or imipenem [12]. Difficult-to-treat (DTR) resistance in *Pseudomonas aeruginosa* and *Acinetobacter* spp. was defined as resistance to all first-line antibiotics, including beta-lactams (carbapenems included) and fluoroquinolones [13].

## 2. Materials and Methods

Data were retrieved from the institutional Laboratory Information System, which stores all routine clinical laboratory results generated at the Department of Clinical Microbiology and ATB Centre, General University Hospital in Prague. The dataset covered the period from 1 January 2024, to 31 December 2024. All positive blood culture (BC) results containing any ESCAPE pathogens (excl. *Clostridioides difficile)—Enterococcus faecium*, *Staphylococcus aureus*, *Acinetobacter species*, *Pseudomonas aeruginosa*, and Enterobacterales—were retrieved from the system. For each positive BC or sepsis episode, the corresponding surveillance culture records were subsequently reviewed to verify concordance between screening outcomes and blood culture findings.

The data were collected from departments with consistent periodical microbiological screening—Acute Care Department (AC) and Haemato-Oncology Department (HO)—and compared. Surveillance cultures were obtained at the admission and then twice weekly from urine, rectal swabs, and specimens from the upper respiratory tract (throat, nose) and/or the lower respiratory tract (bronchial aspirates).

At least two blood cultures were drawn when clinical signs suggestive of sepsis (e.g., fever, hypotension, tachycardia) and/or laboratory indicators of sepsis (e.g., leucocytosis, elevated inflammatory markers) occurred together with proven or suspected infection. Culturing was performed using the Bactec™ system (BD and Company, Franklin Lakes, NJ, USA).

Samples flagged as positive were subsequently inoculated onto solid culture media, such as Columbia blood agar (OXOID/Thermo Scientific, Waltham, MA, USA) and UriSelect™ 4 (Bio-Rad, Hercules, CA, USA); bacteria cultured was identified with matrix-assisted laser desorption and ionisation–time of flight (MALDI-TOF; Bruker, Billerica, MA, USA). Additionally, antibiotic susceptibility testing was performed according to the EUCAST methodology based on the results of Gram staining [12]. For the disc diffusion method, Mueller–Hinton agar and antibiotic discs (both: OXOID/Thermo Scientific, Waltham, MA, USA) were used. When necessary—such as in cases of unclear results or when disc diffusion was not adequate—the minimum inhibitory concentration was determined using the gradient strip method, with Mueller–Hinton agar for Etest^®^ and gradient strips (both: bioMérieux SA, Marcy l’Étoile, France).

For the evaluation of the study outcomes, concordance was defined as the detection of the bacterium causing a BSI episode in a surveillance culture obtained within ≤7 days prior to the BSI episode, exhibiting an identical antimicrobial susceptibility pattern, thereby reflecting identification of the organism before its isolation from blood cultures.

### 2.1. Statistical Analysis

Data were extracted and subsequently anonymized prior to analysis. Microbiological findings from surveillance cultures and BSI episodes were categorised according to specimen type, pathogen species, and antimicrobial resistance profile. Descriptive statistical analysis was performed, with results expressed as absolute numbers and percentages to determine the incidence of individual microorganisms across different specimen types. Concordance between pathogens detected in surveillance cultures and those isolated from blood cultures during BSI episodes was assessed and reported as proportions. Given the descriptive nature of the study, no inferential statistical testing was performed.

### 2.2. Data

In the Acute Care Department (AC) in 2024, 6046 surveillance cultures were performed: 1189 rectal swabs, 1216 urine samples, 2471 upper respiratory tract (URT) samples, and 1170 lower respiratory tract (LRT) samples from total of 542 patients. In the Haemato-Oncology Department (HO) in 2024, 7267 surveillance cultures were performed: 2449 rectal swabs, 2175 urine samples, and 2643 URT samples from total of 251 patients.

## 3. Results

### 3.1. Surveillance Cultures

From all surveillance cultures from AC and HO, MDROs accounted for 5% and 6.56%, respectively. The proportions of relevant resistance phenotypes are shown in Table 1. In AC, 86.89% of MDROs were ESBL-producing Enterobacterales (ESBL-E): *E. coli* 24.92%, *K. pneumoniae* 31.15%, and other Enterobacterales 30.82%. In HO, 81.55% of MDROs were ESBL-E: *E. coli* 46.33%, *K. pneumoniae* 19.29%, and other Enterobacterales 15.93%. This indicates that, relative to all MDROs, HO had roughly double the proportion of ESBL-producing *E. coli* if compared to AC. Conversely, the proportions of ESBL-producing *K. pneumoniae* and other ESBL-E were approximately half in HO compared to AC.

Table 2 summarises the incidence and resistance phenotypes of pathogens detected in surveillance cultures. ESBL production varied by screening sample type and was generally more frequent in rectal swabs. At HO, ESBL-producing *Escherichia coli* was detected in 23.93% of rectal swabs and 9.52% of upper respiratory tract (URT) samples, while in AC the corresponding proportions were 40% and 3.33%, respectively. For *Klebsiella pneumoniae*, ESBL rates at HO were similar in rectal swabs and URT samples (52.94% vs. 46.67%); however, at AC, ESBL production was more common in rectal swabs (47.6%) than in URT (8.33%) or lower respiratory tract (LRT) samples (22.47%).

Carbapenemase production (CP) was rare in the study setting. Among 819 *Escherichia coli* detections in rectal swabs at HO, 10 (1.22%) were CP, half of which produced metallo-beta-lactamase (MBL). Although 14.71% of *Klebsiella pneumoniae* detections were carbapenem-resistant, none produced carbapenemases.

Among Gram-positive bacteria, notable detections were observed in *Enterococcus faecium* and *Staphylococcus aureus*. The incidence of *E. faecium* in screening samples differed markedly between HO and AC. At HO, *E. faecium* was detected in 25.97% (*n* = 636) of 2449 rectal swabs, of which 6.45% were vancomycin-resistant and 6.45% linezolid-resistant (only 1 strain showed resistance to both). In contrast, at AC, *E. faecium* was identified in only 1.77% (*n* = 21) of 1189 rectal swabs, with 33.3% (*n* = 7) of detections being vancomycin-resistant.

### 3.2. Blood Cultures

The distribution of ESCAPE pathogens in blood cultures (BC) differed between HO and AC, despite a similar number of BSI episodes. At HO, Gram-negative bacteria accounted for 52.17% and Gram-positive bacteria for 47.83% of ESCAPE detections; at AC, the respective proportions were 80% and 20%. The higher proportion of Gram-positive bacteria at HO was driven by *Enterococcus faecium* (43.48% vs. 5% at AC). Among Gram-negative pathogens, *Escherichia coli* was the most frequent pathogen at HO with 34.78%, whereas at AC the proportions of *E. coli* and *K. pneumoniae* were similar (20% and 25%, resp.). The data are summarised in Table 3.

### 3.3. Comparison Between Blood Cultures and Surveillance Cultures

The study assessed whether pathogens causing BSI episodes were also detected in surveillance cultures. Significant differences were observed between HO and AC, which can be seen in Table 4.

At HO, the majority of ESCAPE pathogens causing BSI episodes were also detected in rectal swabs. All *E. coli* and *K. pneumoniae* BSI detections (100%) were identified in rectal screening samples. Similarly, *E. faecium* was detected in rectal swabs in 81.81% of BSI episodes. Other screening sample types showed limited concordance: only 8.33% of Gram-negative BC detections were additionally found in urine or URT samples. For *E. faecium*, concordance was observed in 18.18% of urine samples and 9.09% of URT samples.

In contrast, at AC, BC detections showed higher concordance with upper and lower respiratory tract (URT and LRT) samples than with rectal swabs. Among Gram-negative pathogens, concordance rates were 56.2% for rectal swabs and 68.75% for URT samples. All *K. pneumoniae* bloodstream detections were concordant with URT samples, compared with 60% with rectal swabs. For *S. aureus*, the highest concordance was observed with URT samples (75%). One BSI episode caused by *Enterococcus faecium* was observed; however, this strain was not detected in any surveillance cultures collected during the study period.

## 4. Discussion

This retrospective study highlights the relevance of routine microbiological surveillance cultures in haemato-oncological patients as well as in patients admitted to intensive care units. The incidence of ESCAPE pathogens—*Enterococcus faecium*, *Staphylococcus aureus*, *Acinetobacter* spp., *Pseudomonas aeruginosa*, and Enterobacterales—detected in surveillance and blood cultures was assessed, and the correlation between colonisation and subsequent bloodstream infection was analysed. Importantly, both resistant and susceptible isolates were included, as information on susceptibility patterns is crucial for clinical decision-making and initial antimicrobial therapy in these vulnerable patient populations.

Colonisation with multidrug-resistant organisms (MDROs) has been repeatedly shown to predict invasive infection, mainly in immunocompromised and critically ill patients. Surveillance cultures therefore represent a potential tool for early risk stratification and for guiding initial antimicrobial therapy at the onset of sepsis [14,15,16].

Beyond the detection of resistant pathogens, surveillance cultures also provide valuable information on susceptible flora, which may support de-escalation strategies and help avoid unnecessary broad-spectrum antimicrobial use. For example, in a Dutch study of 372 patients, the authors found that the use of surveillance cultures to guide empirical antimicrobial therapy was associated with a potential reduction in carbapenem use of 82.8% [17].

Colonisation is recognised as a risk factor for infection; however, colonisation data for various MDROs are not routinely considered when selecting antibiotic treatment, and the literature reports heterogeneous results regarding the predictive value of colonisation screening for informing therapeutic decision-making [18]. The previously mentioned Dutch study demonstrated a high negative predictive value of surveillance cultures for BSI caused by third-generation cephalosporin-resistant Enterobacterales—99.1% [17]. In contrast, a review from the United States reported considerably lower positive predictive values for ESBL-producing Enterobacterales, ranging from 40% to 50%, limiting the potential for carbapenem-sparing strategies [19].

In the Czech Republic, screening practices show substantial heterogeneity, and it remains unclear in which clinical settings screening is consistently implemented. According to a study from Switzerland, public hospitals generally reported higher rates of institutional screening, particularly for ESBL-producing Enterobacterales (89%), and carbapenemase-producing Enterobacterales (CPE, 83%). Despite the decreasing trends in *S. aureus* resistance, methicillin-resistant *S. aureus* (MRSA) screening was carried out across all public facilities. In contrast, private hospitals showed more heterogeneous practices, with lower overall institutional screening and a greater proportion of facilities performing no screening in some instances—for example, 26% for ESBL and 22% for CPE [20].

The observed heterogeneity in screening practices likely reflects the absence of universally accepted guidelines defining target patient populations, screened pathogens, and sampling frequency. As a result, the clinical utility of surveillance cultures remains inconsistently evaluated across institutions [21]. However, the existence of guidelines does not guarantee their adoption. In 2020, the Italian Association of Clinical Microbiologists (AMCLI) published recommendations for laboratory-based surveillance of MDROs in long-term care facilities [22]. A follow-up evaluation by the same working group in 2024 revealed that 87.5% of facilities had not implemented these measures [23].

This study demonstrated a relatively low incidence of multidrug-resistant organisms (MDROs) in surveillance cultures. Overall, MDROs accounted for 6.56% of detections at Haemato-Oncology Department (HO) and 5.0% at Acute Care Department (AC). Despite this low overall incidence, multidrug-resistant Gram-negative bacteria predominated, representing 81.55% of MDRO detections at HO and 95.09% at AC, with Enterobacterales being the most frequently identified group (81.55% and 86.89%, respectively).

These findings partially align with those reported by Torres et al., who likewise observed a high prevalence of MDR Gram-negative bacteria. However, their study reported a substantially higher proportion of non-fermenting Gram-negative rods, particularly *P. aeruginosa* and *Stenotrophomonas maltophilia*, compared with Enterobacterales (55.7% vs. 34.2%) [8]. In contrast, no MDR non-fermenting Gram-negative rods were detected at HO in our study, and only low proportions were observed at AC (*P. aeruginosa*, 7.87%; *Acinetobacter* spp., 0.33%), with no *S. maltophilia* identified in either department. This discrepancy may reflect regional epidemiological differences. Non-fermenting Gram-negative bacteria are typically associated with the healthcare environment and its infrastructure; therefore, rigorous environmental monitoring and regular cleaning in our setting may limit their spread [24].

Our findings indicate a low incidence of Gram-positive MDROs (18.45% at HO and 4.92% at AC), which is broadly consistent with the results reported in the Spanish study (10.1%) [8].

The incidence of MDRO colonisation varies considerably across different healthcare settings. An Italian study reported that the majority of MDROs detected in rectal swabs were vancomycin-resistant enterococci (VRE; 64%) and carbapenemase-producing *K. pneumoniae* (23%) [25]. Similarly, a different Spanish study focusing on carbapenemase-producing organisms classified colonised patients as cases and found that 49.3% were colonised with carbapenemase-producing *K. pneumoniae*, 36.6% with *Acinetobacter baumannii*, and 23.9% with *E. coli*. In contrast, control patients in the same study were predominantly colonised with methicillin-resistant *S. aureus* (MRSA; 27.3%) and MDR *P. aeruginosa* [26].

Recent evidence underscores the growing clinical relevance of colonisation with carbapenem-resistant Enterobacterales (CRE). In a study by Karakosta et al., 31.1% (*n* = 1323) of screened patients (total *n* = 4252) were colonised with CRE in their setting. Notably, 15.6% of colonised patients subsequently developed BSI caused by the same pathogen, with KPC-producing Enterobacterales predominating [27]. Similarly, in an earlier study from 2014, Giannella et al. reported that 7.8% of 1813 carriers of CR *K. pneumoniae* developed BSI caused by the same pathogen [28]. By comparison, in the present study setting, only 19 of 793 included patients (2.4%) were colonised with carbapenem-resistant Enterobacterales, and none developed BSI during the study period.

According to the European Centre for Disease Prevention and Control (ECDC), the incidence of carbapenem-resistant *K. pneumoniae* in invasive isolates (blood cultures) in Greece remained high in 2024 at 60.2% and has been relatively stable over the past decade (69.7% in 2023, 66.3% in 2020, and 61.9% in 2015). In contrast, substantially lower rates have been reported in the Czech Republic, with incidences of 2.2% in 2024, 1.5% in 2023, 0.5% in 2020, and 0.3% in 2015. This pronounced disparity highlights marked regional differences in the epidemiology of carbapenem-resistant bacteria across Europe [29].

Taken together, these findings highlight the importance of setting-specific microbiological screening strategies that include surveillance cultures. Such approaches provide valuable insight into local epidemiological patterns of MDRO colonisation and resistance, thereby supporting informed infection control measures and potentially facilitating the selection of appropriate initial antimicrobial therapy [30].

Overall, the rate of BSI episodes (caused by ESCAPE pathogens) in high-risk patients in our cohort was lower than that reported in several previously mentioned studies: 4.79% of patients developed at least one episode of BSI during their hospitalisation. This finding may, at least in part, be influenced by the implementation of systematic and regular microbiological screening using surveillance cultures. Early identification of colonisation may allow for timely infection control measures and closer clinical monitoring, potentially reducing the progression to overt BSI. In haemato-oncological patients, the use of pre-emptive antimicrobial therapy based on colonisation status may further contribute to this effect. Together, these factors may help explain the comparatively low incidence of BSI observed in our study population.

The aim of this study was to assess whether the causative agents of BSI episodes belonging to the ESCAPE group could be predicted using routine microbiological screening with surveillance cultures. In the Haemato-Oncology Department, Gram-negative pathogens causing BSI were detected in rectal swabs in most cases (83.33%), with *E. coli* and *K. pneumoniae* identified in all cases. This finding may be explained by gastrointestinal mucosal injury in haemato-oncological patients, related to both the underlying disease and its treatment, which can lead to translocation of intestinal flora into the bloodstream [31,32].

In contrast, in the Acute Care Department, a higher concordance was observed between blood culture results and respiratory screening samples, including throat swabs and lower respiratory tract specimens. For *K. pneumoniae*, concordance reached 100%, underscoring the relevance of respiratory samples for the detection of Gram-negative colonisation. This pattern may reflect the clinical characteristics of acute care patients, who frequently experience respiratory failure and require various forms of respiratory support, potentially facilitating bacterial colonisation of the respiratory tract and subsequent entry into the bloodstream [33].

Torres et al. analysed surveillance cultures in patients with ventilator-associated pneumonia (VAP) and reported that 87.5% of MDR Gram-negative pathogens causing BSI were also detected in pharyngeal swabs, with Enterobacterales accounting for the majority of concordant isolates (62.5%) [33].

This study has several limitations that should be considered. First, it was conducted at a single centre, so the results may not be directly generalizable to other settings. To account for variability, two departments with different patient populations were included. Second, its retrospective design may introduce bias and relies on existing medical records, which can be incomplete or inconsistent. Additionally, only patients with available surveillance and blood cultures were included, and variability in sampling practices and laboratory methods may have influenced pathogen detection and resistance profiling. Furthermore, given the descriptive nature of this study and the relatively low number of BSI episodes, formal statistical testing was not performed. Despite these limitations, the study provides valuable insights into the incidence of ESCAPE pathogens and the correlation between colonisation and BSI in high-risk patients. By including both resistant and susceptible isolates, our findings contribute to a better understanding of the potential role of surveillance cultures in guiding initial therapy and informing antimicrobial stewardship strategies in vulnerable patient populations.

## 5. Conclusions

This retrospective study demonstrates that the distribution of bacteria detected in surveillance cultures varies substantially by specimen type and clinical setting in patients experiencing BSI episodes, underscoring the importance of a setting-specific approach to microbiological surveillance. In both haemato-oncology and acute care units, surveillance cultures provided relevant information on colonisation patterns and resistance phenotypes associated with subsequent BSI. These findings support the routine use of surveillance cultures as a complementary tool for early risk stratification and infection control in high-risk patient populations. Incorporating surveillance culture data into sepsis management may facilitate more timely and targeted initial antimicrobial therapy.

## Figures and Tables

**Table 1:** Multidrug-resistant bacteria in the Haemato-Oncology and Acute Care Departments, by phenotype.

		HO		AC	
		*n*	%	*n*	%
Total MDR		477	100	305	100
*Escherichia coli*	ESBL	221	46.33	76	24.92
	CR	14	2.94	2	0.66
	CP	11	2.31	2	0.66
*Klebsiella pneumoniae*	ESBL	92	19.29	95	31.15
	CR	22	4.61	11	3.61
	CP	0	0	9	2.95
Other Enterobacterales	ESBL	76	15.93	94	30.82
	CR	2	0.42	2	0.66
	CP	1	0.21	2	0.66
*Pseudomonas aeruginosa*	DTR	0	0	24	7.87
*Acinetobacter* spp.	DTR	0	0	1	0.33
*Enterococcus faecium*	VRE	44	9.22	7	2.30
	LRE	45	9.43	0	0
	VRE + LRE	1	0.21	0	0
*Staphylococcus aureus*	MRSA	0	0	8	2.62

AC, Acute Care Department; CP, carbapenemase-producing; CR, carbapenem-resistant; DTR, difficult-to-treat; ESBL, extended-spectrum beta-lactamase; HO, Haemato-Oncology Department; LRE, linezolid-resistant enterococci; MDR, multidrug-resistant bacteria; MRSA, methicillin-resistant *Staphylococcus aureus*; VRE, vancomycin-resistant enterococci.

**Table 2:** ESCAPE bacteria detected from surveillance samples.

		HO			AC			
		Rectal Swab	Urine	URT	Rectal Swab	Urine	URT	LRT
		% (*n*)	% (*n*)	% (*n*)	% (*n*)	% (*n*)	% (*n*)	% (*n*)
*Escherichia coli*	Total	100 (819)	100 (121)	100 (21)	100 (165)	100 (47)	100 (90)	100 (57)
	ESBL	23.93 (196)	19.01 (23)	9.52 (2)	40 (66)	10.64 (5)	3.33 (3)	3.51 (2)
	CR	1.59 (13)	0.83 (1)	0	1.21 (2)	0	0	0
	CP	1.22 (10)	0.83 (1)	0	1.21 (2)	0	0	0
	MBL	0.61 (5)	0	0	1 (0.6)	0	0	0
*Klebsiella pneumoniae*	Total	100 (136)	100 (12)	100 (30)	100 (105)	100 (23)	100 (156)	100 (89)
	ESBL	52.94 (72)	50 (6)	46.67 (14)	47.6 (50)	52.17 (12)	8.33 (13)	22.47 (20)
	CR	14.71 (20)	0	6.67 (2)	6.67 (7)	0	0.64 (1)	1.12 (1)
	CP	0	0	0	5.71 (6)	0	0.64 (1)	1.12 (1)
	MBL	0	0	0	4.76 (5)		0.64 (1)	1.12 (1)
Other Enterobacterales	Total	100 (235)	100 (17)	100 (23)	100 (130)	100 (32)	100 (266)	100 (156)
	ESBL	29.36 (69)	35.29 (6)	4.35 (1)	37.6 (49)	25 (8)	7.14 (19)	11.54 (18)
	CR	0.85 (2)	0	0	0	0	0.38 (1)	0.64 (1)
	CP	0.43 (1)	0	0	0	0	0.38 (1)	0.64 (1)
	MBL	0	0	0	0	0	0.38 (1)	0.64 (1)
*Pseudomonas* *aeruginosa*	Total	100 (45)	0	100 (13)	100 (203)	100 (22)	100 (145)	100 (101)
	CR	20 (9)	0	23.08 (3)	10.34 (21)	40.91 (9)	10.34 (15)	16.83 (17)
	DTR	0	0	0	2.46 (5)	22.7 (5)	4.14 (6)	7.92 (8)
	MBL	0	0	0	0.49 (1)	0.49 (1)	0	0
*Acinetobacter* spp.	Total	100 (1)	0	100 (2)	100 (7)	0	100 (17)	100 (10)
	CR	0	0	0	14.2 (1)	0	0	0
	DTR	0	0	0	14.2 (1)	0	0	0
*Enterococcus faecium*	Total	100 (636)	100 (52)	100 (15)	100 (21)	100 (2)	100 (28)	100 (35)
	VRE	6.45 (41)	0	20 (3)	33.33 (7)	0	0	0
	LRE	6.45 (41)	5.77 (3)	6.67 (1)	0	0	0	0
	VRE + LRE	0.16 (1)	0	0	0	0	0	0
*Staphylococcus aureus*	Total	100 (2)	0	100 (8)	100 (2)	100 (1)	100 (113)	100 (43)
	MRSA	0	0	0	0	0	6.19 (7)	2.33 (1)

AC, Acute Care Department; CP, carbapenemase producing; CR, carbapenem-resistant; DTR, difficult-to-treat; ESBL, extended-spectrum beta-lactamase; HO, Haemato-Oncology Department; LRE, linezolid-resistant enterococci; LRT, lower respiratory tract; MBL, metallo-beta-lactamase;; MRSA, methicillin-resistant *Staphylococcus aureus*; URT, upper respiratory tract; VRE, vancomycin-resistant enterococci.

**Table 3:** ESCAPE bacteria detected from blood cultures.

	HO	AC
	% (*n*)	% (*n*)
Total	100 (23)	100 (20)
Gram-negative bacteria	52.17 (12)	80 (16)
*Escherichia coli*	34.78 (8)	20 (4)
*Escherichia coli* ESBL	4.35 (1)	0
*Klebsiella pneumoniae*	8.7 (2)	25 (5)
*Klebsiella pneumoniae* ESBL	4.35 (1)	10 (2)
*Klebsiella aerogenes*	4.35 (1)	5 (1)
*Serratia marcescens*	4.35 (1)	5 (1)
*Enterobacter cloacae* complex	0	5 (1)
*Enterobacter cloacae* complex ESBL	0	5 (1)
*Pseudomonas aeruginosa*	0	15 (3)
Gram-positive bacteria	47.83 (11)	20 (4)
*Enterococcus faecium*	43.48 (10)	5 (1)
*Enterococcus faecium* LRE	8.7 (2)	0
*Staphylococcus aureus*	4.35 (1)	15 (3)
*Staphylococcus aureus* MRSA	0	10 (2)

AC, Acute Care Department; ESBL, extended-spectrum beta-lactamase; HO, Haemato-Oncology Department; LRE, linezolid-resistant enterococci; MRSA, methicillin-resistant *S. aureus.*

**Table 4:** Percentage concordance of ESCAPE pathogens between blood cultures and surveillance cultures.

	HO			AC			
	Rectal Swab	Urine	URT	Rectal Swab	Urine	URT	LRT
	% (*n*)	% (*n*)	% (*n*)	% (*n*)	% (*n*)	% (*n*)	% (*n*)
Gram-negative bacteria	83.33 (10)	8.33 (1)	8.33 (1)	56.2 (9)	37.5 (6)	68.75 (11)	37.5 (6)
*Escherichia coli*	100 (8)	12.5 (1)	0	50 (2)	100 (4)	25 (1)	0
*Escherichia coli* ESBL	100 (1)	100 (1)	0	0	0	0	0
*Klebsiella pneumoniae*	100 (2)	0	0	60 (3)	20 (1)	100 (5)	60 (3)
*Klebsiella pneumoniae* ESBL	100 (1)	0	0	100 (2)	50 (1)	100 (2)	50 (1)
*Klebsiella aerogenes*	0	0	0	0	100 (1)	100 (1)	100 (1)
*Serratia marcescens*	0	0	100 (1)	0	0	0	0
*Enterobacter cloacae* complex	0	0	0	50 (1)	0	100 (2)	100 (2)
*Enterobacter cloacae* complex ESBL	0	0	0	100 (1)	0	100 (1)	100 (1)
*Pseudomonas aeruginosa*	0	0	0	100 (3)	33.33 (1)	66.66 (2)	0
Gram-positive bacteria	81.81 (9)	0	0	0	0	(3)	(1)
*Enterococcus faecium*	81.81 (9)	18.18 (2)	9.09 (1)	0	0	0	0
*Enterococcus faecium* LRE	18.18 (2)	0	9.09 (1)	0	0	0	0
*Staphylococcus aureus*	0	0	0	0	0	75 (3)	25 (1)
*Staphylococcus aureus* MRSA	0	0	0	0	0	50 (2)	25 (1)

AC, Acute Care Department; ESBL, extended-spectrum beta-lactamase; HO, Haemato-Oncology Department; LRE, linezolid-resistant enterococci; LRT, lower respiratory tract; MRSA, methicillin-resistant *Staphylococcus aureus*; URT, upper respiratory tract.

## Data Availability

The original contributions presented in the study are included in the article. Further inquiries can be directed to the corresponding author.
